# Bibliographical

**Published:** 1886-05

**Authors:** 


### BIBLIOGRAPHICAL.

WÖRTERBUCH DER BACTERIENKUNDE (BACTERIOLOGICAL DICTIONARY). By DR. W. D. MILLER, Professor in the Dental School of the University of Berlin. Stuttgart: Ferd. Enke, 1886.

Dr. Miller's contributions to bacteriological literature have been numerous and of great importance, and, we are proud to say, have been made largely through the pages of the INDEPENDENT PRACTITIONER. To his indefatigable industry we are now indebted for a glossary of the words and terms used in bacteriological parlance, which will prove of the greatest service to all students of the subject. It is by no means an extensive work, comprising only forty-three pages; nevertheless, we find concise definitions of all the peculiar terms and expressions used by bacteriologists, as well as brief descriptions of bacterial forms. For instance, taking at random: *Aspergillus fumigatus,* we find "A pathogenic species of aspergillus, with white mycelium and green spores; found in the lungs of men and animals, and in the human nose."

*Leptotherix buccalis,* "Long thread-like forms, which assume a blue color when treated with iodine and acids; found in the mouths of men and animals. Properties not yet known. Incorrectly held, by some, as the cause of dental caries."

*Micrococcus of acute yellow atrophy of the liver.* "Doubtful."

*Syphilis bacilli.* "3-7 micro-millimeters long, cells of various shapes (comma, S-form, spirilli, etc.), found in syphilitic secretions, chancres, etc. Alleged cause of syphilis. (Lustgarten.)"

These extracts will serve to show the character of the work, which, we are informed, is only the precursor of a much more pretentious volume, of similar but enlarged scope, now in preparation.

The errors in the present one are rather those of omission. Thus *spaltpilze* is defined, but its counterpart, *schimmelpilze,* is not. As a glossary for the use of those only reasonably familiar with German, it would seem more suitable to insert the description of the organisms now generally known in all lands as the *staphylococcus pyogenes albus* (seu aureus) under this term, rather than the clumsy German equivalents, *weisser* or *gelber traubencoccus.* Nevertheless, these trifles do not detract from the value of the work. We shall look forward with eagerness to the appearance of Dr. Miller's larger work.

MIGNONETTE. An Ideal Love Story, by Sangrée (Mrs. Linda Sangrée Allen). New York: G. W. Carleton & Co., 1886.

This delightful volume of three hundred and twenty-five pages has a peculiar attractiveness and charm for dentists, the authoress being the widow of the well-known Dr. William H. Allen, of New York City, formerly Professor of Operative Dentistry in the New York College of Dentistry, who died in October, 1882. Many will remember the charming woman who sometimes attended with him the meetings of the American Dental Association, and it is to her that we are indebted for the production of this book. The dedication contains a tender tribute to the memory of the lamented Dr. Allen.

Although this is the first published work of its gifted authoress, nowhere in its pages will the reader be reminded of the fact. The diction is smooth and flowing, the narrative connected and easy, and the interest maintained to the end. There is a vein of peculiar tenderness and grace running through the whole volume, as of a refined and gentle nature softened by affliction and exalted by sorrow. In all that interprets the language of the heart, it is delicately sweet and tender. This is especially true of the chapter which contains the love-making between Paul and Ethel. Even the incidents that are more commonly a part of French than American novels are invested with a grace that makes them seem natural, and its analysis of the sentiments of the heart is at times profound.

The book will make a charming ornament for the centre-table of the dentist's office, and we heartily commend it to the consideration of every one of our readers.

C. ASH & SONS' DENTAL CATALOGUE, 1886.

This is a catalogue of the different dental goods manufactured and sold by C. Ash & Sons, of London. This firm is becoming better known in America, and were it not for the high protection tariff levied on foreign goods, they would probably become as famous here, as in England and on the continent.

The book is a very beautiful specimen of the printer's art. Some of the illustrations of instruments and manufactures are really artistic in design and execution. The colored illustration of dental rubbers shows twenty-four different tints, and must have been passed through the press fifty times, to give all the colors, with the lettering and varnish. It is a beautiful specimen of color printing.

